# Spinal cerebrospinal fluid leak in the context of pars interarticularis fracture

**DOI:** 10.1186/s12883-020-01740-1

**Published:** 2020-04-29

**Authors:** Tommy Lik Hang Chan, Robert Cowan, Nada Hindiyeh, Syed Hashmi, Bryan Lanzman, Ian Carroll

**Affiliations:** 1 Division of Headache and Facial Pain, Department of Neurology & Neurological Sciences, Stanford University, Palo Alto, CA USA; 2 Department of Rad/Neuroimaging and Neurointervention, Stanford University, Palo Alto, CA USA; 3 Department of Anesthesia, Stanford University, Palo Alto, CA USA

**Keywords:** Spinal cerebrospinal fluid leak, Spinal CSF leak, Intracranial hypotension, Pars interarticularis fracture

## Abstract

**Background:**

Spinal cerebrospinal fluid (CSF) leak can lead to intracranial hypotension and is an important differential diagnosis to consider in patients with sudden-onset chronic daily headaches. Pars interarticularis (PI) fracture is a potential rare cause of suspected spinal CSF leak.

**Methods:**

This is a retrospective case series of 6 patients with suspected spinal CSF leak evaluated between January 2016 and September 2019. All patients received a magnetic resonance imaging (MRI) of the brain with and without gadolinium, MRI whole spine and full spine computed tomography (CT) myelogram. Targeted epidural patches with fibrin sealant were performed. Treatment response at return visit (3 months post-patch) was documented.

**Results:**

Six patients (4 females, 2 males) were diagnosed with a suspected spinal CSF leak and PI fracture. Mean age at the time of headache onset was 39 years old, and a range from 32 to 50 years old. Mean time to targeted epidural patches with fibrin sealant was 4.5 years. All 6 patients had PI fractures identified on CT myelogram and received targeted epidural patches with fibrin sealant at the site of the PI fracture. All patients had significant improvement in their headache intensity.

**Conclusion:**

Our study highlights: 1) the importance of PI fracture as a possible culprit of suspected spinal CSF leak in patients with intracranial hypotension; 2) the added benefit of CT imaging for detecting bony abnormalities such as fractures in patients with intracranial hypotension; and 3) the successful treatment of suspected spinal CSF leak when targeting the fracture site.

## Background

Spinal cerebrospinal fluid (CSF) leak can lead to intracranial hypotension and is an important differential diagnosis to consider in patients with sudden onset chronic daily headaches. This entity can often be confused with other chronic headache disorders, such as new daily persistent headache and chronic migraine. The most common clinical manifestation of a spinal CSF leak is orthostatic headache (worse upright) [1]. However, there is a wide variation of clinical presentation in headache arising from a spinal CSF leak including non-positional headache [1–4], second half of the day headache [5], exertional headache [3, 6], cough headache [7], thunderclap headache [8–10], and paradoxical headache (in which symptoms are worse when supine) [11–15].

In addition to orthostatic headache, patients with spontaneous intracranial hypotension (SIH) can experience a broad clinical spectrum of symptoms, including cochleovestibular dysfunction [16, 17], cranial nerve palsies, visual blurring, upper limb symptoms, encephalopathy, autonomic dysfunction, and cognitive dysfunction [18–20]. This is presumptively due to sagging of the brain causing significant traction, distortion or compression of anchoring and supporting structures [21]. Several imaging modalities have been utilized to aid in the diagnosis of spinal CSF leak including magnetic resonance imaging (MRI) of the brain with and without gadolinium, MRI whole spine, radioisotope cisternography, magnetic resonance myelogram, computed tomography (CT) myelogram or digital subtraction myelogram, each with proposed individual benefits and drawbacks [22, 23].

Spinal CSF leak is commonly precipitated by traumatic events such as motor vehicle accidents, sports injuries, and medical procedures such as dural or epidural punctures [22]. However, only 30% of patients with SIH can identify an inciting event or trauma, with the majority of patients unable to trace the onset of symptoms to any unusual circumstances at all [24]. SIH may arise in an area of a pre-existing dural sac weakness secondary to connective tissue disorders or spinal conditions such as herniated disc or spondylotic spurs [22]. However, there is no available medical literature highlighting osseous fracture as a potential cause of suspected spinal CSF leak, including pars interarticularis (PI) fracture. PI fracture is a stress fracture involving the posterior vertebral arch. This injury occurs in the lower lumbar region, most often at L5 and S1 [25]. Discovery of a PI fracture is often an incidental imaging finding in an asymptomatic patient or in an adolescent athlete involved in sports requiring repetitive lumbar loading in extension and rotation [26]. In this setting, patient may present with acute or insidious onset lower back pain. We suspect PI fracture can rarely be a potential cause of suspected spinal CSF leak. In this retrospective case series, we identified PI fractures in patients with suspected spinal CSF leak and their clinical outcomes were reported.

## Methods

Six cases of suspected spinal CSF leak secondary to PI fracture were identified retrospectively. All patients were evaluated between January 2016 to September 2019 at the Stanford CSF leak headache clinic. All cases were presented at the neuroradiology/headache neurology CSF leak conference with headache specialists, anesthesiologists and neuroradiologists in attendance. All patients received an MRI of the brain with and without gadolinium, MRI whole spine and full spine CT myelogram. No bedside lumbar puncture was performed. These studies were interpreted by board-certified neuroradiologists. All targeted epidural patches with fibrin sealant were performed by a team member board-certified in Headache, Anesthesiology, and Pain Medicine (IC). Treatment response at return visit (3 months post-patch) was documented based on a pain intensity scale from 0 to 10, 10 being the most severe. This study received IRB approval (eProtocol 52,298, IRB 61, Registration 4947) from Stanford University.

### Statistical analysis

This is a descriptive case series study only, so no statistical analysis was utilized.

## Results

**Fig. 1:**
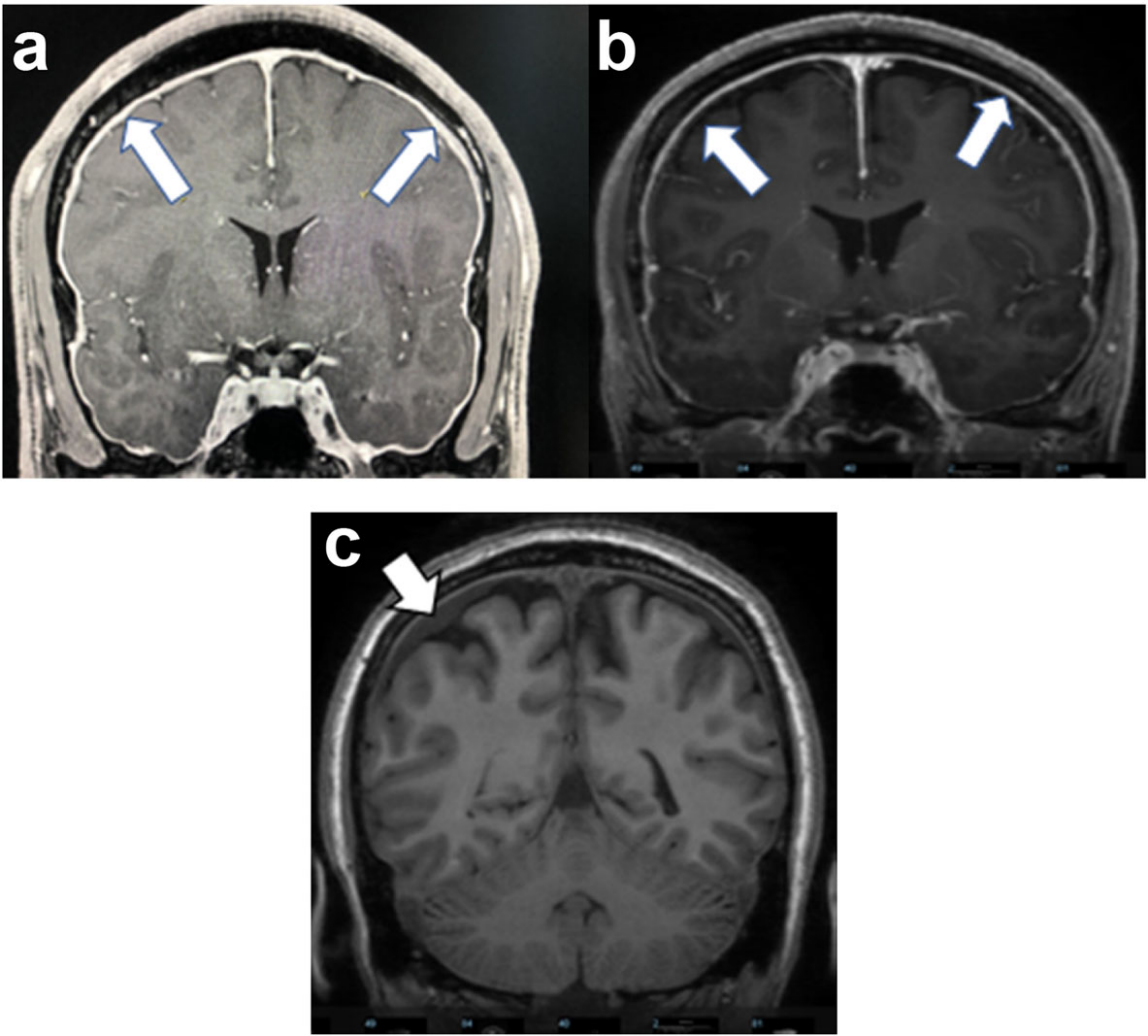
**a** Patient 1 **b** Patient 2: Magnetic Resonance Imaging with gadolinium (Coronal T1 Spoiled Recalled Gradient-Echo) demonstrated diffuse pachymeningeal enhancement (arrows), a feature of intracranial hypotension. **c:** Patient 2: Magnetic Resonance Imaging (Coronal T1) demonstrated subdural collections (arrow), a feature of intracranial hypotension

Six patients (4 females and 2 males) had suspected spinal CSF leak based on their clinical and/or radiological findings. All 6 patients complained of daily unremitting orthostatic headaches. Patient 3, 4, and 5 had migraine features (throbbing/pounding, moderate to severe intensity, worse with activity, photophobia, phonophobia, and nausea) with their headaches. Patient 1, 2, and 6 had severe occipital pressure pain with their headaches. They had a number of concomitant symptoms including blurry vision, tinnitus, ear fullness, altered taste, vertigo, neck pain, interscapular pain, autonomic dysfunction and/or cognitive dysfunction (Table 1). Mean age at the time of onset of headache was 39 years old, with a range from 32 to 50 years old. Mean time to targeted epidural patches with fibrin sealant was 4.5 years. All 6 patients had limited therapeutic response to pharmacological treatment listed in Table 1. Four patients had an MRI brain reported as normal and 2 patients had radiological features suggestive of intracranial hypotension (Patient 1 and 2 had pachymeningeal enhancement [Fig. 1a, b], patient 2 had subdural collections [Fig. 1c]). All 6 patients had PI fracture identified on CT myelogram. Patient 1 and 6 recalled an inciting event (after hiking and after a mechanical fall respectively) with onset of headache within 24 h. Patient 1 complained of lower back pain. Four patients had contrast nerve sleeves enhancement at multiple spinal levels and patient 1 had epidural fluid collection on CT myelogram. Opening pressure was not recorded in 5 patients and 1 patient (Patient 5) had an opening pressure measured at 18 cm H20 from the CT myelogram. The mean number of interventions before targeted treatment at the PI fracture site was 3.8. The clinical and radiological characteristics for the 6 documented patients are included in Table 2, while treatment method and response are documented in Table 3. All 6 patients had an improvement (Greater than 50% reduction) in their headache intensity at their return visit (3 months post-patch) documented based on a pain intensity scale from 0 to 10, 10 being the most severe after treatment targeted at the PI fracture site (Table 3). All 6 patients indicated a subjective improvement of their concomitant symptoms and reported a reduction in their analgesics use. Patient 1 and 4 developed suspected rebound intracranial hypertension 6 months after treatment with reverse postural headache (worse when supine), improved with diuretics and/or carbonic anhydrase inhibitors.

**Table 1:** Patient Medical History

Patient	Past Medical History	Concomitant Symptoms	Pharmacological Treatments
1	None	Neck stiffness, tinnitus, interscapular pain, lower back pain, autonomic dysfunction and anxiety	Ibuprofen
			Oxycodone
2	None	Blurry vision, tinnitus, ear fullness, vertigo and cognitive dysfunction	Oxycodone
			Ibuprofen
3	Ehlers-Danlos Syndrome	Tinnitus, vertigo, neck pain, autonomic and cognitive dysfunction	Sertraline
	Migraine		Sumatriptan
			Naproxen
4	Depression	Blurry vision, neck pain, tinnitus, ear fullness, altered taste, interscapular pain, autonomic and cognitive ysfunction	Amlodipine
	Hypertension		Amitriptyline
	Migraine		Naproxen
5	Prior C5/6 discectomy Depression	Blurry vision, neck pain, tinnitus, ear fullness, altered taste, interscapular pain, autonomic and cognitive dysfunction	Amitriptyline
			Acetaminophen
6	Corneal transplant	Neck pain, tinnitus, ear fullness, difficulty swallowing and cognitive dysfunction	Acetaminophen

**Table 2:** Patient Characteristics

Patient # (Age Gender)	Age of onset	Inciting event	Orthostatic headache (Worse upright)	MRI head (with and without gadolinium)	MRI spine	CT myelogram	Previous treatment^a^ (# of patches)
1 (35 M)	32	Post-hiking	Yes	Diffuse pachymeningeal enhancement	Central disc protrusion at T7/8, T11/12	Bilateral L5 PI #	4
						Diffuse epidural fluid collection in the whole spine	
					Epidural fluid collection extending from L1-L5		
2 (52F)	50	None	Yes	Diffuse pachymeningeal enhancement	Severe grade 3 L4/5 spondylolisthesis	Bilateral L4 PI #	3
						L4/5 anterolisthesis	
				Subdural collections		Mildly prominent perineural spaces at bilateral C7-T1, right T7/8, T8/9, bilateral T9/10	
						Small left S2 perineural cyst	
3 (38F)	34	None	Yes	Normal	Mild degenerative changes in the C and L spine	Bilateral L5 PI #	4
						Multi-disc degeneration from C3-C6	
						Small amount of contrast seen in multiple perineural sleeves throughout the spine	
4 (49F)	39	None	Yes	Normal	C3/4, C4/5, C6/7 disc bulges	Bilateral L3 PI #	2
						Grade 1 L3/4 anterolisthesis	
					L3/4 anterolisthesis	Nerve root sleeve opacification at the left C6/7, C7/T1, L5/S1 and right T12/L1	
					S1 Tarlov cyst		
5 (38F)	34	None	Yes	Normal	Prior discectomy at C5/6	S1 PI # (right)	3
						Bilateral nerve root sleeves at C5/6, C6/7, C7/T1, T7/8, T9/10, T10/11 and all L levels	
6 (49 M)	45	Fall	Yes	Normal	Normal	Bilateral L5 PI #	7
						Calcified osteophyte on left L5/S1 facet join	

*C* Cervical *T* Thoracic *L* Lumbar *S* Sacral PI #: Pars Interarticularis fracture

^a^All patients received conservative treatment plus a combination of the following: blind epidural blood patches, targeted blood patches and/or fibrin sealant

**Table 3:** Treatment Response

Patient	Treatment method (epidural patch with fibrin sealant, paramedian approach) ^**a**^	Treatment response	
		Pre-treatment^**b**^	Post-treatment^**b**^ (3 months post-patch)
1a)	Bilateral L4/L5, right L5/S1	Upright: 3	Upright: 0
		Supine: 2	Supine: 0
2	Bilateral L4/L5	Upright: 8	Upright: 4
		Supine: 2	Supine: 1
3	Bilateral L4/L5, left L5/S1	Upright: 8	Upright: 2
		Supine: 0	Supine: 0
4b)	Bilateral L3/4	Upright: 8	Upright: 4
		Supine: 4	Supine: 2
5	Right L4/L5, right L5/L6	Upright: 7	Upright: 3
		Supine: 0	Supine: 0
6	Bilateral L5/S1	Upright: 4	Upright: 1
		Supine: 3	Supine: 0

^a^3 ml of fibrin sealant to each site

^b^Pain intensity scale: 0–10, 10 being the most severe

a) At 6 months – suspected rebound intracranial hypertension managed with acetazolamide 250 mg twice daily

b) At 6 months – suspected rebound intracranial hypertension managed with amiloride 10 mg and furosemide 60 mg daily

### Case illustration (Patient 1)

**Fig. 2:**
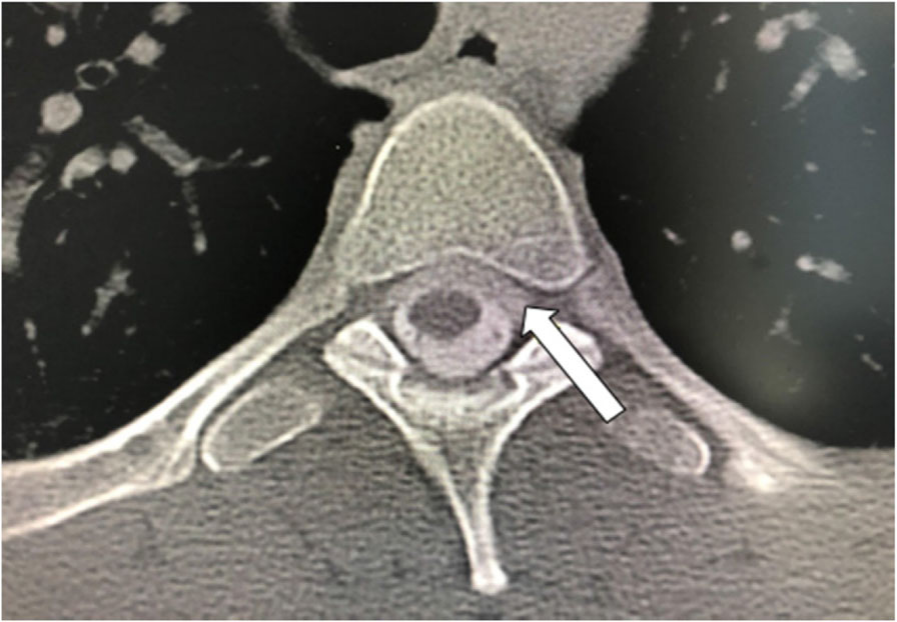
Computed Tomography Myelogram (axial view) demonstrated a ventral cervicothoracic epidural CSF collection (arrow) extending from the mid cervical spine to mid thoracic level (Patient 1)

**Fig. 3:**
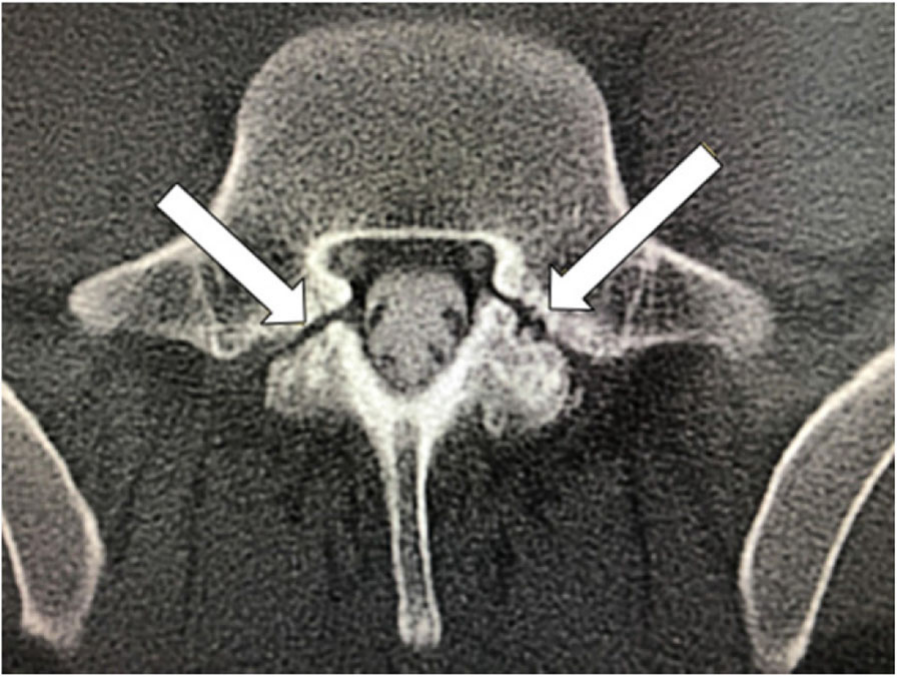
Computed Tomography Myelogram (axial view) demonstrated bilateral L5 pars interarticularis fractures (arrows) (Patient 1)

A 32-year-old male developed a severe positional headache following a hiking trip. The headache location was occipital in nature. He also complained of tinnitus, interscapular pain, lower back pain, autonomic dysfunction and anxiety shortly after the onset of his orthostatic headache. Ibuprofen and oxycodone provided no relief. His past medical history was unremarkable. Neurological examination was unremarkable. MRI of the brain with and without gadolinium demonstrated diffuse pachymeningeal enhancement (Fig. 1a). Full spine CT myelogram revealed a ventral cervicothoracic epidural fluid collection extending from the mid cervical spine to approximately T8 (Fig. 2). He underwent 4 patches over a span of 2 years: 2 non-directed lumbar epidural blood patches, bilateral transforaminal T6–7 and right transforaminal T10–11 epidural blood patches with fibrin sealant (3 ml each site), left paramedian C7-T1 and right T2-T3 paramedian interlaminar blood patches with catheters threaded under fluoroscopic guidance to the ventral epidural space (10 ml and 5 ml respectively). The 2 non-directed lumbar epidural blood patches provided transient and mild relief, but the subsequent 2 targeted patches provided no relief. Finally, the bilateral L5 PI fracture site (Fig. 3) was targeted with bilateral paramedian L4–5 and right paramedian L5–S1 epidural patches with fibrin sealant (3 ml each site). In contrast with his previous patches, he noted resolution of his orthostatic headache with pain intensity dropped from 3 to 0 upright, on a scale of 0 to 10, 10 being the most severe at his return visit (3 months post-patch). He discontinued his ibuprofen and oxycodone intake completely. He later returned to his full-time employment with minimal concomitant symptoms.

## Discussion

All 6 patients suffered from chronic daily headaches and were diagnosed with suspected spinal CSF leak based on their clinical and/or radiological findings. Spinal CSF leak may resolve spontaneously or through conservative measures such as bed rest, oral hydration, generous caffeine intake and use of an abdominal binder [22, 23]; however, this was not seen in our patients. They did not respond to pharmacological treatment, conservative measures and failed multiple interventions including non-directed epidural blood patches and epidural patches targeted at levels outside of the PI fracture site, such as at the level of the contrast enhanced nerve sleeves and extradural collection. This highlights that in many cases of spinal CSF leak, it is imperative to identify the site of spinal CSF leak for targeted treatment with epidural patches to maximize response rate [23]. These cases highlight the difficulty in successfully identifying a target site.

The exact location of spinal CSF leak is often unknown. MRI brain may suggest signs of intracranial hypotension (subdural collections, pachymeningeal enhancement, venous engorgement, pituitary hyperemia and brain sagging), but these have no localizing value [23]. Spinal imaging such as MRI or CT myelogram can confirm a leak based on contrast in the extradural space or nerve sleeves, but may be falsely localizing [27]. Our patients had targeted low volume (3 ml each site) epidural patches with fibrin sealant at the site of the PI fractures with significant treatment response (Greater than 50% reduction in pain intensity) at their 3 month return visit, which suggests the PI fractures may be the culprit for their suspected spinal CSF leak. Previous reports have demonstrated intracranial hypotension or pseudomeningocele as potential complications from other orthopedic injuries including spondylolisthesis and vertebroplasty [28, 29]. PI fractures were detected from the CT myelogram and were missed on the MRI spine in 5 out of the 6 patients. Our cases illustrate an added advantage of CT myelogram in the workup of suspected spinal CSF leak for osseous pathology [30].

Patient 1 demonstrated a transient and mild relief from his non-targeted lumbar epidural patches, which suggests a possibility that prior treatments were coincidentally injected at or near the site of PI fracture since the lumbar region is often a standard location for non-directed epidural blood patches. Limited information was available from chart review on the other patients’ prior treatment response.

Patients 1 and 4 developed suspected rebound intracranial hypertension 6 months post-patch based on a change in their headache characteristics (headache worse lying supine). This information was relayed to our center from their primary providers. This illustrates a potential risk of spinal CSF leak treatment long term. Our patients had a prolonged clinical course before the PI fracture was identified as the suspected leak site. It is possible that contributed to an increased risk of rebound intracranial hypertension [31].

As for the PI fracture, the treatment is usually nonsurgical (rest and bracing). Surgery (laminectomy or posterior lumbar fusion) may be required if symptoms persist or remain bothersome [25]. Patient 1 complained of lower back pain, which could be a direct symptom of PI fracture, however his subjective improvement after the patch argued against the PI fracture being the cause and lower back pain is not an uncommon symptom in SIH [20].

### Limitations

Some limitations were seen with this study: Two out of the 6 patients (Patient 1 and 2) met the diagnostic criteria of “headache attributed to low CSF pressure” as per the International Classification of Headache Disorders Third Edition [32]. The other 4 patients had suspected CSF leak based on supportive clinical and radiological findings, such as orthostatic headaches, enhancing nerve sleeves and/or favorable response to treatment. All cases were presented at the neuroradiology/headache neurology CSF leak conference and the diagnosis of suspected CSF leak was agreed upon a panel of headache specialists, anesthesiologists and neuroradiologists. Opening pressure was not measured in 5 patients and patient 5 had a normal opening pressure recorded [33]. Certainly, a detection of CSF pressure < 6 cm H2O supports the diagnosis of CSF leak, however, it is not uncommon to have a normal opening pressure with CSF leak, especially in chronic CSF leak patients. A normal opening pressure does not rule out a CSF leak [23]. All patients reported subjective improvement with their concomitant symptoms at their return visit, but not quantified. Long term follow-up (beyond 3 months) data are not available since our center is a tertiary referral center and our patients returned back to their primary and referring providers for subsequent follow-up. This is a retrospective case series limited by lack of control subjects and small population size. It is also subject to selection and information bias.

## Conclusion

The diagnosis and management of spinal CSF leak causing SIH remains a challenge, especially in cases where the site of suspected CSF leak is difficult to detect. There are limitations to each imaging modality and often a combination is required to confirm and localize a CSF leak. Our study highlights: 1) the importance of PI fracture as a possible culprit of suspected spinal CSF leak in patients with intracranial hypotension; 2) the added benefit of CT imaging for detecting bony abnormalities such as fractures in patients with intracranial hypotension; and 3) the successful treatment of suspected spinal CSF leak when targeting the fracture site. If no clinical improvement is observed after adequate conservative measures (bed rest, oral hydration, generous caffeine intake and use of an abdominal binder) or non-targeted epidural patches in the context of a non-localizing spinal CSF leak, further testing may be warranted to explore an osseous pathology, such as occult fractures. No conclusions can be drawn regarding the frequency with which PI fractures are complicated by CSF leaks. Future research in a larger cohort of patients is required to explore the likelihood of detecting PI fracture in patients with non-localizing spinal CSF leak and to identify any risk factors associated with both PI fracture and spinal CSF leak. Long term follow-up data are required to explore the effectiveness and potential complication (i.e rebound intracranial hypertension) from targeted patches to the fracture site.

## Data Availability

The material analyzed during the current study are available from the corresponding author on reasonable request. Permission was obtained to access the patient data and the data was de-identified upon data collection.

## Abbreviations

CSF: Cerebrospinal Fluid
CT: Computed Tomography
MRI: Magnetic Resonance Imaging
PI: Pars Interarticularis
SIH: Spontaneous Intracranial Hypotension
